# Listen, Learn, Like! Dorsolateral Prefrontal Cortex Involved in the Mere Exposure Effect in Music

**DOI:** 10.1155/2012/846270

**Published:** 2012-03-28

**Authors:** Anders C. Green, Klaus B. Bærentsen, Hans Stødkilde-Jørgensen, Andreas Roepstorff, Peter Vuust

**Affiliations:** ^1^Department of Psychology, Aarhus University, Jens Christian Skous Vej 4, 8000 Aarhus C, Denmark; ^2^MR Research Centre, Aarhus University Hospital, Brendstrupgårdsvej 100, 8200 Aarhus N, Denmark; ^3^Center of Functionally Integrative Neuroscience, Aarhus University Hospital, Nørrebrogade 44, Building 10G, 5th Floor, 8000 Aarhus C, Denmark; ^4^Royal Academy of Music, Skovgaardsgade 2C, 8000 Aarhus C, Denmark

## Abstract

We used functional magnetic resonance imaging to investigate the neural basis of the mere exposure effect in music listening, which links previous exposure to liking. Prior to scanning, participants underwent a learning phase, where exposure to melodies was systematically varied. During scanning, participants rated liking for each melody and, later, their recognition of them. Participants showed learning effects, better recognising melodies heard more often. Melodies heard most often were most liked, consistent with the mere exposure effect. We found neural activations as a function of previous exposure in bilateral dorsolateral prefrontal and inferior parietal cortex, probably reflecting retrieval and working memory-related processes. This was despite the fact that the task during scanning was to judge liking, not recognition, thus suggesting that appreciation of music relies strongly on memory processes. Subjective liking per se caused differential activation in the left hemisphere, of the anterior insula, the caudate nucleus, and the putamen.

## 1. Introduction

The ability of music to cause emotional reactions and enjoyment in listeners is undoubtedly crucial to its existence. Previous neuroimaging studies [1–4] focused on *how* the brain reacts to pleasant music, leaving open *why *people like the music they do. Music preferences differ widely between individuals, groups, and cultures. These differences cannot be due solely to qualities inherent in the music itself, since the same piece may be a “hit” to one person, but a “flop” to the next. Psychomusicological studies, for example, [5, 6] have pointed to the *mere exposure effect* (MEE) as an important part of the explanation. The MEE means that simple exposure to a novel, neutral stimulus by itself increases liking for it [7, 8]. Accordingly, studies of music preference generally find that liking increases with exposure [5, 6], a phenomenon which is utilised in practice by the music and advertising industries.

While the MEE is a well-established behavioural phenomenon, we know little about its biological basis. Only a single neuroimaging study, by Elliott and Dolan [9], has so far focused specifically on it. That study used subliminal visual stimuli, and it showed particular involvement of the right lateral prefrontal cortex in implicit memory expressed in preference judgements, indicating this as a key brain area related to the MEE.

As for the MEE of music, our study is the first to examine its neural basis. We examined supraliminal (rather than subliminal) stimuli, which are probably most relevant to everyday music listening. A learning phase familiarised participants with a number of melodies, where the amount of exposure to subsets of the melodies was varied systematically in order to induce different levels of implicit learning. Subsequently, they were scanned using functional magnetic resonance imaging (fMRI), while performing a liking judgement of the melodies, succeeded by a recognition test.

We hypothesized that the right lateral prefrontal cortex would play a role in the MEE for music, as it did for visual stimuli [9], that is, show differential activation as a function of prior exposure. However, given the absence of prior studies on the subject, additional brain areas could be involved. Regarding perception of stimulus novelty, on the other hand, we expected involvement of the medial temporal (including hippocampal) region based on evidence from the study by Elliott and Dolan [9], as well as others, for example [2, 10–12]. Concerning activations related to subjective liking, we hypothesized the participation of limbic and paralimbic areas previously linked to positive judgements of music, such as insula, striatum, anterior cingulate, and orbitofrontal cortex [1–4]. Additionally, it was of interest to us if the results would support the valence lateralization model [2, 13, 14], that is, that neural responses to positively judged melodies would be left-lateralized.

## 2. Materials and Methods

### 2.1. Stimuli and Participants

#### 2.1.1. Stimuli

**Figure 1:**
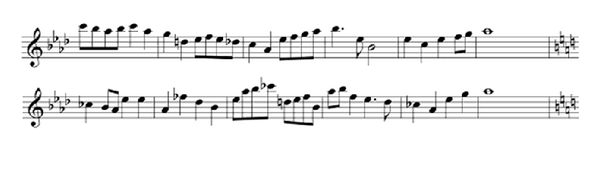
Stimulus examples. Two examples of melodies from the stimulus material; one in Ab-major (top) and one in Ab-minor (bottom).

30 single-voice melodies of 13 s duration each were composed for the study, see Figure 1 for two examples. Melodies were digital sequences in both piano and guitar versions, quantised and generated in Cubase SX, postprocessed in Adobe Audition.  10 melodies were in the key of A flat, 10 in E, and 10 in C. The 10 melodies in each key were further balanced between major and minor mode, resulting in a total of six different melody types (see [15] for the report on neural correlates of musical mode). Other parameters of the melodies were kept identical, including ambitus (a 10th), tempo (80 beats per minute), and amplitude. 

 As we wished to study exposure effects, we used unknown melodies to eliminate the possibility of preexperimental knowledge of the stimuli. In composing these melodies we aimed to strike a balance between experimental control and musical-ecological validity. Melodies were composed adhering to the guidelines mentioned above, in order to render them comparable, thus avoiding differences in neural activation solely on the basis of variation of superficial features, such as timbre or tempo. On the other hand, it was also necessary to induce a certain level of variety and song-like qualities in the melodies, in order to make them stand out from each other, and be appreciated as individual pieces of music. We used supraliminal stimuli (as opposed to for example Elliott and Dolan [9]), since music naturally unfolds over seconds and minutes, rather than milliseconds, and since listeners in real life are in fact most often aware (at least potentially) of the music they are listening to. 

Additionally, six melodies for the learning phase were made along identical guidelines as above, but incorporating a single note detuned by 50 cents (half a semitone). The melodies containing a detuned note were not used in the scanning part of the experiment. 

An ascending chromatic scale of uniformly distributed intervals (minor seconds), but otherwise with similar characteristics as the melodies, was also employed in the fMRI part of the experiment. 

#### 2.1.2. Stimulus Grouping

A preexperimental liking rating of the melodies was done by 49 volunteers, who did not participate in the fMRI experiment itself. This rating was carried out in order to divide the melodies into five groups containing six melodies each, so that the mean liking for each melody group would be as equal as possible. Each group consisted of one melody from each of the six possible melody types. The mean liking ratings for the five groups were 2.95, 2.97, 2.97, 2.94, and 2.98, respectively, on a scale from 1 to 5 (overall mean 2.96, SD = 0.51). 

#### 2.1.3. Participants

21 healthy right-handed (by self-report) volunteers, 12 female, mean age 27.3 (range 20–33) participated in the scanning experiment. Participants were nonmusicians, but musically adept, as indicated by pitch and rhythm tests, as used at the Royal Academy of Music, Aarhus, Denmark. In these tests, participants were asked to reproduce progressively more complex melodies and rhythms, by singing (pitch) or clapping (rhythm). The ethical committee of Aarhus county approved the study, and participants gave their informed consent prior to their inclusion in the study. 

### 2.2. Procedure and fMRI Data Analysis

#### 2.2.1. Procedure

For each participant, the experiment incorporated five phases (A–E); two outside the scanner, followed by two in the scanner, and lastly a debriefing and musical ability test outside the scanner. See Table 1 for an overview. Phases occurred in immediate succession. 

Phases A and B utilised five different pseudorandomised scenarios of stimulus sequences, four of these were used four times each, and the last one five times (for a total of 21, corresponding to the number of participants). Total randomisation was avoided to ensure that melodies of the same key and mode would not succeed each other, and that the occurrence of different stimulus types were uniformly separated (by employing a split-halves method). In the scanning phases C and D, stimulus sequences were pseudorandomised for each participant individually in order to avoid the amplification of possible sequence effects.

**(A) Baseline Rating and Training Phase**

Participants listened to 18 melodies over headphones, that they would hear again in phase B. They rated each melody immediately after listening to it, in a two-second gap of silence between melodies, on a 1–5 scale (1: least liked, 5: most liked).

**(B) Learning Phase**

Participants listened again to the 18 melodies, which were divided into three different groups of six. The melodies were played in pseudorandomised order, either once, seven or 31 times each, depending on which group they belonged to. This meant that participants would have heard a given melody either two, eight, or 32 times, at the conclusion of phase B, depending on scenario (see above).

Due to the large number of stimulus presentations in this phase, resulting in a total duration of about an hour, three measures were taken to aid the concentration of participants. First, participants were asked to press a button as quickly as possible to the occurrence of detuned notes in the six special melodies that would appear five times each throughout phase B. Second, instrument sound in this phase alternated between piano and guitar, in order to help differentiate one melody from the next (the guitar versions of the melodies were not used for the scanning sessions). Lastly, participants were allowed a five minute break halfway through the learning phase.

**(C) Liking Scanning Phase**

In the scanner, participants listened to these 18 melodies again once each, along with six further melodies that had not been heard previously (for a total of 24). The 24 melodies thus belonged to one of four categories designated F0, F2, F8, and F32, based on the number of previous repetitions in the given scenario. This resulted in a total of 126 trials (melody presentations) for each exposure category (six melodies in each group, times 21 subjects). Participants were instructed to visually fixate on a crosshair on a screen, listen carefully to the stimuli over the headphones, and immediately after each melody to rate how well they liked it on a scale from 1 (least liked) to 5 (most liked), using a response box with one button for each finger. The scale and rating procedure was identical to the one in phase A, and thus well known to participants. Stimulus sequences further included six repetitions of the chromatic scale, and a number of 2–8 s silences in between, yielding jittered interstimulus intervals to allow more exhaustive sampling of the fMRI signal.

**(D) Memory Phase**

Identical to the preceding phase, except for two important differences. First, a further six new melodies were introduced (bringing the total to 30 in five categories, designated F0, F1, F2, F8, F32). Second, the task for the participants was to judge their certainty of having heard the melody before (1: certain that melody has not been played, 5: certain that melody has been played). Again, there were 126 total trials in each exposure category. The rating was done with participants in the scanner, as for phase C, in order to ensure that the recognition rating was done in exactly the same physical context as the previous liking rating, and to have the two ratings as close to one another as possible in time.

**(E) Debriefing Interview and Musical Tests Phase**

Following the fMRI scan, participants were debriefed, interviewed about their experiences during the experiment, and pitch and rhythm tests were performed to confirm a basic level of musical ability (a 22nd subject was excluded from the data analysis on these grounds).

#### 2.2.2. fMRI Image Acquisition and Data Analysis

Scanning was performed on a Signa Excite 1.5 Tesla MR scanner (General Electric Medical Systems, Milwaukee, WI, USA). T1-weighted anatomical images were acquired, and functional images were obtained using a gradient-echo echoplanar imaging sequence; TR = 2700 ms, TE = 40 ms. 34 axial slices covering the whole brain were acquired; 5 mm thickness (no gap) with an in-plane resolution of 3.44 × 3.44 mm (64 × 64 matrix). 

FMRI data analysis of the data from the scanning phase C was done with SPM5 (Institute of Neurology, London, UK). Data preprocessing consisted in realignment to the first image and unwarping, coregistration with the T1-weighted images, and slice timing. Subsequently, segmentation of gray and white matter, together with spatial normalisation, was performed with the unified segmentation approach [16]. During the spatial normalisation process images were re-sampled into 2 mm isotropic voxels. Images were spatially smoothed using a 10 mm full width at half maximum Gaussian kernel. 

Periods with acoustic stimuli were modelled with a hemodynamic response function, whereas periods of subjective ratings after each stimulus were not modelled. The subjective liking rating, and the objective exposure frequency category (F0, F2, F8, or *F*32), pertaining to each melody, were entered into the model as parametric modulations. Experimental effects were estimated with a general linear model [17]. Contrasts were set up to test effects of subjective liking, based on individual subject ratings, and for effects of stimulus exposure frequency. Second-level random effects analyses yielded statistical parametric maps. These were thresholded at *P* < 0.001, uncorrected at voxel level. Only clusters of more than 10 voxels were reported in order to minimise the risk of including spurious activations. Voxel coordinates were transformed from Montreal Neurological Institute to Talairach space (http://imaging.mrc-cbu.cam.ac.uk/imaging). Anatomical locations were established using mainly Talairach and Tournoux [18] and the Talairach Client software [19]. 

#### 2.2.3. Behavioural Data Analysis

To analyse the effects of exposure on liking and recognition, respectively, one-factor repeated measures analyses of variance (ANOVA) were carried out on the data from phase C and D (see Table 1 and Section 2.2.1). The factor was exposure, and conditions were the varied amounts of previous exposure. These are termed “F0” through “F32,” as described in Subsection 2.2.1. To ascertain which conditions prompted any significant effect observed, post hoc pairwise comparisons were done, using LSD (Least Significant Difference). Reaction times for the liking and recognition ratings were analysed using the same methods. 

## 3. Results

### 3.1. Behavioural Results

#### 3.1.1. Liking Rating Results

**Figure 2:**
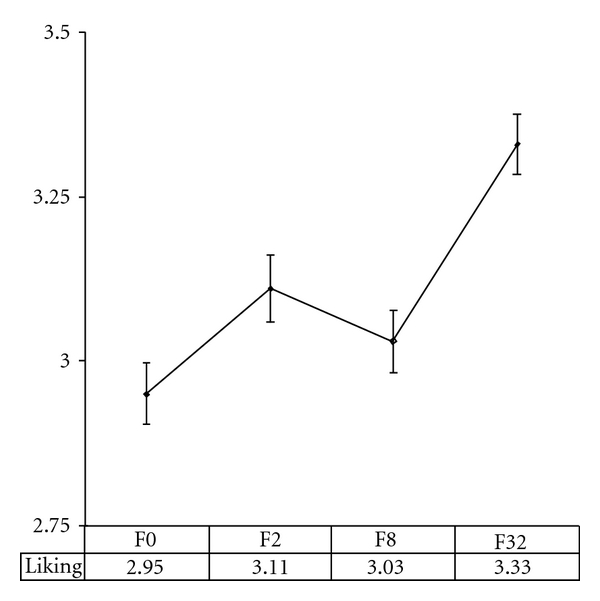
Liking and previous exposure. Mean liking rating as a function of previous exposure (melodies heard 0, 2, 8, or 32 times previously). Error bars represent standard error.

The mean liking ratings for melodies heard 0, 2, 8, and 32 times by the end of the learning phase, are shown in Figure 2 (the groups are termed F0 (Frequency 0), F2, etc.). The ratings were obtained during the fMRI scan (i.e., phase C; refer to Section 4 for experimental procedure). Repeated measures ANOVA showed a statistically significant effect of exposure on liking rating, *F*  (3,60) = 2.784, *P* = 0.048. Pairwise comparisons of means showed that liking for the *F*32 group was significantly higher than for the F0 group, *t*  (20) = −2.312, *P* = 0.032. None of the other pairwise comparisons was statistically significant (although the F8 to F32 difference was borderline significant, *P* = 0.051). Adjusting for the baseline (phase A) rating of each melody did not change this pattern of findings. 

Reaction times (RTs) for the liking ratings differed as a function of presentation frequency, as shown by a repeated measures ANOVA, *F*  (3,60) = 3.842, *P* = 0.014. Pairwise comparisons of means revealed that subjects responded faster to the F32 group than to the other groups, *F*0 versus *F*32 : *t*  (20) = 3.08, *P* = 0.006; *F*2 versus *F*32 : *t*  (20) = 2.103, *P* = 0.048; *F*8 versus *F*32 : *t*  (20) = 2.541, *P* = 0.019. The actual temporal difference was small; 1.65 secs. for the *F*32 group versus 1.80 secs on average for the three other groups, yielding a 0.15 secs. mean difference. RT also differed slightly as a function of subjective liking rating, *F*  (4,44) = 3.278, *P* = 0.019. RT for “1” and “5” ratings were on average respectively 0.09 secs and 0.13 secs faster than the overall mean for liking ratings. 

#### 3.1.2. Recognition Rating Results

**Figure 3 fig3:**
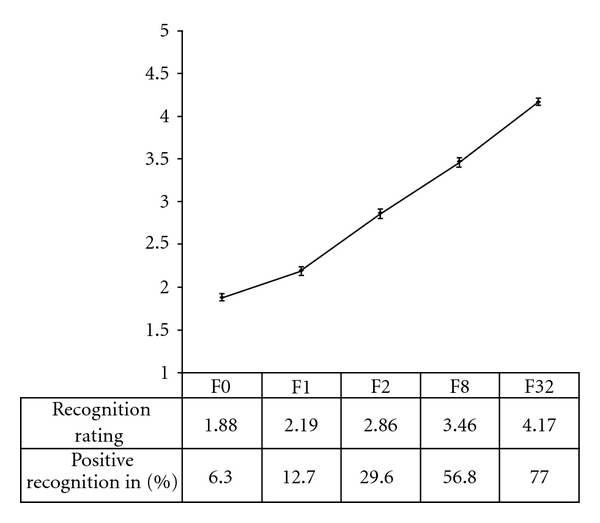
Recognition and previous exposure. Mean recognition ratings as a function of previous exposure (melodies heard 0, 1, 2, 8, or 32 times previously). Error bars represent standard error. The bottom line of the table shows the percentage of the total melodies in each exposure group that were rated as positively recognised, that is, given a rating of 4 or 5.

Mean recognition ratings for melodies heard 0, 1, 2, 8, and 32 times before are shown in Figure 3. Repeated measures ANOVA showed a statistically highly significant effect of exposure on recognition rating, *F*  (3.42,68.42) = 71.734, *P* < 0.001 (the sphericity assumption was not met, so Huynh-Feldt correction was applied). Pairwise comparisons of means revealed that every pair of means differed significantly; paired samples *t*-test, *P* < 0.05. 

RT for memory ratings did not differ significantly as a function of exposure, *F*  (3.06,61.20) = 1.098, *P* = 0.358 (the sphericity assumption was not met, so Huynh-Feldt correction was applied). 

### 3.2. fMRI Results

#### 3.2.1. Effect of Subjective Liking on Brain Activity

**Figure 4:**
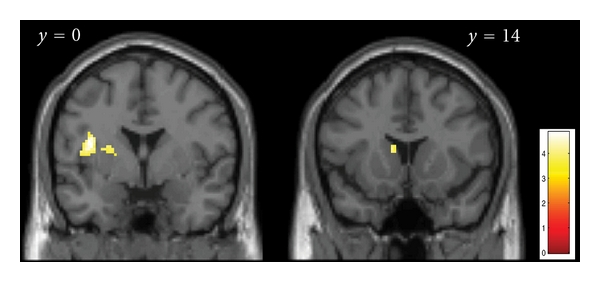
Visualization of brain regions related to liking. Coronal sections with statistical activation maps of the liking effect, at *y* = 0 (left), and *y* = 14 (right). Areas showing increased activity with increased liking were (left section): left anterior insula, rolandic operculum, and putamen; (right section): caudate nucleus. Random effects analysis; *P* < 0.001, uncorrected, only clusters of more than 10 voxels included. The left side of each section corresponds to the left hemisphere.

Brain regions showing increased activity as a function of subjective liking rating (irrespective of presentation frequency) were all located in the left hemisphere and included a cluster in the anterior part of the insula (BA 13), extending into the rolandic operculum (Table 2 and Figure 4). Further activations were found in the dorsal striatum, namely, in the putamen, and in the body of the caudate nucleus. The opposite contrast, of areas showing decreased activity as a function of liking rating, did not result in any significant activation. 

#### 3.2.2. Effect of Exposure Frequency on Brain Activity

**Figure 5:**
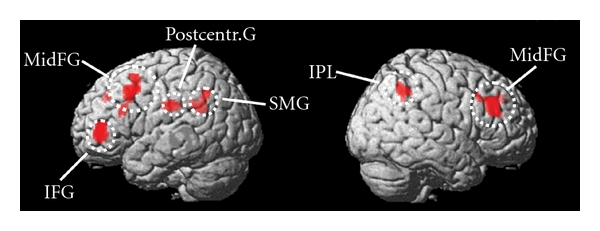
Visualization of brain regions related to exposure effect. Surface rendering of the statistical activation map of the exposure effect in listening to melodies (areas showing increased activity with increased previous exposure). Random effects analysis; *P* < 0.001, uncorrected, only clusters of more than 10 voxels included. IFG: inferior frontal gyrus; IPL: inferior parietal lobe; MidFG: middle frontal gyrus; Postcentr. G: postcentral gyrus; SMG: supramarginal gyrus. Leftmost rendering is of the left hemisphere.

The contrast based on the amount of previous exposure to the melodies showed activity increases in dorsolateral prefrontal cortex (DLPFC), namely, in bilateral middle frontal gyri (BA 9), and neighbouring inferior middle/frontal gyri (BA 46). (See Table 2 and Figure 5). Differential activations of the left hemisphere further incorporated activations of the postcentral gyrus (BA 2), and the supramarginal gyrus (SMG) (BA 40). BA 40 of the right inferior parietal lobe was also implicated, as was BA 6 of the left middle frontal gyrus. The opposite contrast, corresponding to an increase in the fMRI signal as a function of stimulus novelty, did not result in any significant activation. 

## 4. Discussion

We have shown that listening to melodies rated as likeable differentially activates deeper brain structures related to emotion processing, all in the left hemisphere, including the anterior insula and the dorsal striatum. A supraliminal mere exposure effect was a contributing factor to how positively melodies were judged, and brain regions subserving this effect, that is, exhibiting increased activation as a function of prior exposure, included the dorsolateral prefrontal cortex (bilateral BA 9 and BA 46). We would like to caution that the results discussed here are based on an analysis uncorrected for multiple comparisons, (see Section 2.2.2) and would therefore benefit from further empirical substantiation. 

### 4.1. Brain Activity Related to Liking Effects

Preceding neuroimaging studies of liking effects in music listening have highlighted the contribution of limbic structures, consistent with the knowledge of their involvement in processing of emotions in general (e.g., [20]). A PET study [1] found several limbic and paralimbic structures to be involved when listeners experienced so-called “chills” from the music. Of these areas, some in particular have been confirmed by one or more of the subsequent studies on responses to pleasant music with PET and fMRI, including the ventral striatum (nucleus accumbens), anterior cingulate, orbitofrontal cortex, and insula [2–4]. There is also evidence suggesting that neural responses to pleasant music in general tend to be more lateralized to the left hemisphere, an effect often termed the “valence lateralization model” [2, 13, 14]. 

Of the brain regions that have been previously implicated in the liking-related network, our study confirmed the involvement of the insula. We also found differential activation of the striatum, more precisely its dorsal part, rather than the ventral part often found in previous studies (see below). We did not confirm the involvement of the anterior cingulate or orbitofrontal areas. This only partial replication of the previously observed liking-related network may relate to the fact, that the melodies composed for our study were relatively short, unknown, computer-recorded single-voice melodies, in order to maximize experimental control. Previous studies [1–4] were based on recorded music excerpts, mostly from the classical repertoire. Activation of the entire liking-related network may be contingent upon using music excerpts varying on a wider range of parameters, such as instrumentation and dynamics, although this could potentially jeopardise stimulus comparability. 

The involvement of the insula, and more specifically its anterior part, was expected based on prior findings, although in our study it was left-lateralized, compared to mostly bilateral in previous studies on pleasant music (ibid.). Menon and Levitin [4] used effective connectivity analysis in their fMRI study to show a strong ventral tegmental area-mediated interaction of nucleus accumbens and the left anterior insula, forming a mesolimbic dopaminergic system that is central to reward processing. The insula is furthermore known to be one of the most important nodal points (together with the hypothalamus) in neural pathways concerned with autonomic, somatic, and emotional functions [21, 22]. On these grounds, Menon and Levitin [4] attributed insula activation during music listening to its role in regulating autonomic and physiological responses to a rewarding and emotionally salient stimulus. While this explanation remains plausible in our study as well, we cannot completely discount a motor response influence, given the slightly faster RT for both the highest and lowest rated melodies (0.09 secs and 0.13 secs faster than the mean, resp.). Although it is not ideal that RT differs across stimulus categories, at least four points indicate that the problem was not of an alarming magnitude in the context of our study. First, the actual difference in RT was quite small. Second, the motor response (button press), occuring after the melody itself, was not included in the data design model. Third, the faster reaction times concerned both extremes of the rating scale, therefore not affecting the subjective liking contrast in a unidirectional way. Fourth, and perhaps most importantly, the fMRI activation map did not appear to be heavily influenced by finger movements, since neither the primary motor nor somatosensory cortices were found to be differentially involved. Moreover, the anterior insula, especially of the left hemisphere has also been implicated in preference judgements of nonauditory stimuli, such as visual (e.g., [23, 24]), and food stimuli [25]. Consequently, with a cautionary note on possible motor-related contamination, the most likely explanation for the anterior insula activity as a function of liking remains its role as a paralimbic emotion- and reward-related region. 

 The rolandic operculum also showed liking-related activation, although the exact location of the activation peak proved difficult, given the relative proximity of functional areas in this region, in conjunction with the spatial resolution limitations of the fMRI method. It is noteworthy that a previous study also indicated rolandic operculum involvement in the perception of pleasant music [3]. In that study, as well as our own, it is possible that the activity of this region was related to sound production planning (wanting to “hum along”), since rolandic opercular areas have been found to be implicated in both overt and covert singing and speaking in previous neuroimaging studies [26–29]. The subjects in our study were instructed to lie still, and consequently the rolandic opercular activity would more likely be related to covert than overt vocalisation. 

 We found activation of the dorsal part of the striatum, namely, the putamen and caudate nucleus. The dorsal striatum has been associated with music before in fMRI studies; the caudate nucleus in a contrast of happy versus neutral music [30], and the putamen in connection with melodic processing [31]. The dorsal striatum has also been implicated in reward processing of various types of stimuli, especially food and drink, for example [25, 32, 33]. This role relies upon the participation of the striatum in the dopaminergic system. Small et al. [33] demonstrated with PET a feeding-induced increase in dopamine release in the dorsal striatum, which at the same time correlated with pleasantness ratings of the food. As for the insula activation, a cautionary note on a possible motor response contamination applies here as well, because the basal ganglia, of which the putamen and caudate nucleus are constituents, are known to play a key role in the planning and modulation of movement. 

We did not find any differential activation in the opposite contrast, that is, related to disliking. Other studies have shown possible neural correlates of unpleasant, dissonant music (e.g., [3, 34, 35]). A plausible reason for our negative finding is that our stimuli were not sufficiently unpleasant to cause significant differential activation based on low subjective liking, as opposed to the dissonant stimuli used in the studies mentioned, which is a musical property known to cause a high degree of unpleasantness. 

Apart from the particular brain structures involved, we also wished to examine whether our results would support the finding of some researchers (e.g., [2, 13, 14]), that neural responses to positively judged melodies are more left-lateralized. The distribution of voxels in our subjective liking contrast was indeed exclusively left-lateralized. This lends some support to a valence lateralization model, but it should be noted that at least some versions of the lateralization model focus on cortical, and more specifically frontal and anterior temporal areas, for example [36], with empirical support concerning music perception by for example an EEG study by Altenmüller et al. [13]. However, later developments of this type of model have included subcortical (limbic and paralimbic) areas as well, for example [37]. Evidence from our own as well as previous studies notwithstanding, there does seem to be a need for further empirical work concerning the lateralization effects of positive judgement of music, as not all studies seem to corroborate such findings, for example [3, 38]. Moreover, it is conceivable that there is a connection between the left lateralization associated with pleasurable melodies, and vocalisation, which in itself could be an explanatory factor behind the observed lateralization effect in this and previous studies (see also the discussion above regarding vocalisation). 

### 4.2. Brain Activity Related to Exposure Effects

Our main hypothesis concerning exposure effects was confirmed. The prefrontal cortex, in particular BA 9 of the middle frontal gyrus, which was also a central finding in the sole prior neuroimaging study of the MEE [9], was one of the two most prominent activation sites in our study. The other was BA 46, which together with BA 9 forms the DLPFC [39, 40]. We also found additional areas of activation (bilateral BA 40, and left BA 2 and 6), which will be discussed afterwards. 

The DLPFC is central to higher-level cognitive operations, such as executive function and memory functions, including retrieval and working memory (e.g., [40]). Working memory plays a crucial role in tasks related to the perception of music, which unfolds over time, since listeners need to maintain the auditory stimuli in short-term memory and relate them to subsequent input, see also [41]. Accordingly, studies on working memory in music perception have shown its relation to dorsolateral and inferior frontal regions, see for example [41, 42] for a review. When listening to a familiar melody, it is conceivable that stored information about it will continuously be compared with the current auditory input, in the working memory system. Platel et al. [43] thus found bilateral activation of mainly BA 9 and 10 of the middle frontal gyri in an episodic music memory task, using PET, and they ascribed this frontal activity to perceptual analysis of the melodies in working memory. Parallel to its role in working memory, the DLPFC has also been widely implicated in memory retrieval, especially when some kind of evaluation of the retrieved information is needed; see [39] for a review. This function of the DLPFC could well have played a role in the observed activity pattern in our study (discussed below in the section on the MEE). It should be noted that DLPFC activation as a function of familiarity is not exclusive to music perception. For example, a PET study by Fletcher and Dolan [44] found that BA 9 of the middle frontal gyrus, extending inferiorly into BA 46, responded to familiar versus novel visually presented words (see also [39] for a review). In sum, the observed DLPFC activation as a function of exposure was expected, based on prior research, and it can best be explained by an increase in automatic retrieval and working memory processes when listening to familiar melodies. 

The parietal activations as a function of exposure can also be tied to memory-related processes, given the evidence on parietal involvement (including BA 40) in memory functions [45–49]. Moreover, the inferior part of BA 40, that is, in particular the SMG, has been described as especially implicated in successful memory retrieval [47, 50]. Subjects in our study certainly exhibited stronger memory traces for melodies heard more often, as shown by the recognition ratings, and this fact may have contributed to the observed inferior parietal activations. (Note, however, that subjects were not asked to explicitly recall melodies for the scanning session reported here, a topic which is discussed below.) Similarly, in the study of the MEE by Elliott and Dolan [9], the left inferior parietal cortex (BA 7 and 40) was associated with retrieval attempt. Furthermore, other neuroimaging evidence has related activation of especially the SMG part of the inferior parietal cortex to music perception [51–54]. Memory functions could very well have played a role in the SMG activity found in those studies. From a connectivity perspective, it is interesting to note that the inferior parietal activations could be connected to the DLPFC activity discussed above, since these two groups of cortical areas form a frontoparietal functional network, which numerous studies have found to be involved in memory and working memory processes in a wide range of cognitive tasks; see for example [46, 55–57]. 

The involvement of the left postcentral gyrus (BA 2), a primary somatosensory area, in conjunction with the premotor cortex (BA 6) activation of the left middle frontal gyrus, could possibly have been related to the button press response itself (which was done using the right hand). RTs differed marginally between melodies heard most often and the rest, and it cannot be completely discounted that this difference had an observable impact on the fMRI signal in this exposure-related contrast. 

We had expected to find participation of medial temporal lobe regions (particularly the hippocampus) in the contrast pertaining to the neural basis of the effect of stimulus novelty—based on findings from studies on both music as well as other stimulus types [2, 9–12]. However, no significant differential activation was observed. A possible reason for this negative finding hinges on the prolonged learning phase, which thoroughly familiarised participants with the general style of the melodic material. Participants may consequently not have perceived previously unheard melodies as sufficiently novel for the medial temporal lobe system to become differentially activated. 

### 4.3. The Mere Exposure Effect in Music: When Memory Influences Appreciation

Previous exposure to melodies did produce an MEE, meaning that participants preferred melodies heard the most often during the learning phase B (Figure 2). Since memory ratings confirmed that previous exposure had a substantial positive impact on the recognition of the melodies as well (Figure 2), it seems highly probable that memory effects leading to this recognition facilitation also played a leading role in the observed liking increase. This interpretation of the behavioural results was supported by the fMRI data, which showed a differential activation increase in memory-related DLPFC and parietal regions, as a function of exposure. Importantly, participants were not actually queried about recognition, or other memory-related tasks, until after the learning phase and scanning (i.e., not until phase D, see Section 2). Participants were thus not aware until this late stage of the experiment that their memory of the melodies would be tested. During the scan (phase C), their task was solely to rate their subjective liking of the melodies. The reported DLPFC and inferior parietal activity therefore most likely reflect unintentional retrieval processes, drawing upon the exposure to the melodies during phase B. Hearing more familiar melodies seems to have automatically engaged the memory system, even though participants did not actively try to remember them. 

Previous behavioural research and theoretical work have often described the MEE as essentially an *implicit* memory phenomenon, for example [5, 58]. The sole previous neuroimaging study of the MEE [9] similarly related the effect to implicit retrieval. Elliott and Dolan (ibid.) incidentally showed a similar neural activation pattern to the one we find, including BA 9. However, while the memory processes of the participants in our study may well have been initiated unintentionally, as well as learned incidentally, these processes cannot be termed implicit, given that the explicit recognition test showed a clear learning effect. This result relates to the fact that we used supraliminal, and not the subliminal stimuli often employed in MEE studies. It is therefore a significant result of the present study that some of the findings from [9] based on subliminal visual stimuli, have now been replicated and expanded upon with relation to supraliminal auditory stimuli. However, the supraliminal nature of our stimuli, resulting in participants being able to consciously recognise melodies when asked to do so (phase D), may in fact have diminished the MEE, since evidence have supported that it is usually stronger for subliminal stimuli [59, 60]. Nonetheless, our stimuli had the benefit of being more relevant to how music is enjoyed in everyday listening. Moreover, some researchers have suggested that explanations of the MEE should not rely crucially on the distinction between explicit and implicit memory [61]. 

A remaining question concerns the underlying reasons for the MEE in music. A few psychological theories do exist about the MEE. The perceptual fluency/attributional model [62, 63] argues that the easy, fluent processing associated with familiar stimuli is misattributed to a positive disposition toward the stimulus itself. Zajonc [64] supports instead the notion that an absence of adverse effects when a stimulus is presented will generate a positive inclination towards it, through the basic mechanism of conditioning. The merits of these theories notwithstanding, we would like to suggest that, especially in the case of music, which is a temporally extended stimulus, exposure effects on liking are highly dependent on *expectancy* (sometimes termed anticipation or prediction). It has been observed in psychomusicological research [65–67] by Meyer and others that the degree of fulfillment of listeners' expectations towards the music greatly influences how it is perceived and appraised. Huron [65] argues that the mere exposure effect should more aptly be considered a “prediction effect.” Behavioural data have supported the notion that implicit learning of melodies does generate expectancy effects in listeners [68, 69]. Apart from these theoretical and behavioural empirical insights about expectancy in music, a magnetoencephalography study [70], see also [71], showed brain networks engaged in so-called predictive coding, see [72, 73], when perceiving music. Future studies will hopefully elucidate the relation between exposure, liking, and expectancy, as well as its neural basis. 

### 4.4. Conclusion

This study investigated the neural correlates of liking and exposure in music perception, as well as their interrelation. Listening to subjectively likeable melodies recruited part of the limbic/paralimbic system also shown by earlier studies. Brain areas showing liking-related activation included the anterior insula, and the dorsal striatum (putamen and caudate nucleus), all left-lateralized, thus providing some support of a valence lateralization model. The mere exposure effect was a contributing factor to how positively melodies were judged, since melodies heard most often during a prescan learning phase were rated as most liked, as well as most recognisable. The brain region subserving this exposure effect was mainly the dorsolateral prefrontal cortex, consistent with the main finding of the only prior neuroimaging study of the mere exposure effect. Our study expanded on the previous by showing the effect for supraliminal auditory stimuli, as opposed to subliminal visual stimuli. Furthermore, our study for the first time provided functional neuroimaging evidence of the mere exposure effect in music, pointing to underlying automatic memory processes being involved. We suggested expectancy as a potentially important factor in these phenomena, requiring further investigation by future studies. 

## Figures and Tables

**Table 1:** Experimental procedure. Overview of the five main phases of the experimental procedure, divided into phases before and after familiarisation with the melodies. For each phase is listed its main purpose, where it took place (in the MRI scanner or not), the central stimuli used, the task of the participant in relation to these stimuli, and a note about which figures and tables relate to each phase.

Exp. phase		Primary purpose	Location (in/outside scanner)	Task	Main stimuli	Related figures/tables
A	Before familiarisation	Familiarise participant with rating task	Outside	Rate liking	18 comparable melodies	Figure 1: stimulus examples
B		Induce graded implicit learning of melodies	Outside	Identify detuned notes	18 melodies, identical to phase A; amount of exposure systematically varied in 3 increments (2, 8, or 32 times)	—
C	After familiarisation	Obtain liking rating for each melody and Record brain activity	Scanner	Rate liking	24 melodies: the 18 from phase A/B, plus 6 new	Figure 2: liking rating as function of exposure Figure 4 and Table 2: brain regions related to liking Figure 5 and Table 2: brain regions related to exposure
D		Obtain recognition rating for each melody	Scanner	Rate recognition	30 melodies: the 24 from phase C, plus 6 new	Figure 3: recognition rating as function of exposure
E		Confirm equal musical abilities among participants	Outside	Pitch and rhythm tests	—	—

**Table 2:** Liking- and exposure-related brain activations. Overview of activations related to liking and to previous exposure when listening to melodies. Talairach coordinates for the peak activation sites, and their *Z* scores, plus the amount of voxels in each cluster are shown. Random effects analysis; *P* < 0.001, uncorrected, only cluster sizes of more than 10 voxels included. WM: white matter; *: areas of activation with an asterisk in the “No. of voxels” column were a part of the cluster reported immediately above it.

Figure		Peak Talairach coord. (xyz)	*Z* score	No. of voxels
	Liking-related activations:			
	L anterior insula (BA 13)	−38 1 13	3.90	119
	L anterior insula (BA 13)	−40 1 17	3.80	*
Figure 4	L anterior insula (BA 13)	−40 5 15	3.60	*
	L rolandic operculum	−46 0 9	3.41	*
	L putamen	−24−3 11	3.46	48
	L caudate nucleus	−10 14 10	3.45	18

	Exposure-related activations:			
	L parietal lobe (WM)	−26−45 26	4.30	184
	L supramarginal gyrus (BA 40)	−30−53 38	3.51	*
	L middle frontal gyrus (BA 9)	−48 19 38	4.24	114
	L inferior frontal gyrus (BA 46)	−36 43 0	4.10	259
	L middle frontal gyrus (BA 46)	−38 43 5	3.85	*
	L postcentral gyrus (BA 2)	−40−20 25	3.96	121
	R middle frontal gyrus (BA 9)	44 32 28	3.64	220
Figure 5	R middle frontal gyrus (BA 46)	42 30 24	3.57	*
	R inferior frontal sulcus (BA 9)	34 17 32	3.21	*
	L medial frontal gyrus (BA 9)	−10 38 31	3.63	26
	R inferior parietal lobe (BA 40)	42−50 43	3.50	108
	R inferior parietal lobe (BA 40)	38−49 37	3.29	*
	L middle frontal gyrus (BA 6)	−32 12 45	3.47	42
	L middle frontal gyrus (BA 6)	−30 14 49	3.45	*
	L frontal lobe (WM)	−32 23 23	3.46	52
	L frontal lobe (WM)	−32 26 15	3.31	*
